# Language Universals Engage Broca's Area

**DOI:** 10.1371/journal.pone.0095155

**Published:** 2014-04-17

**Authors:** Iris Berent, Hong Pan, Xu Zhao, Jane Epstein, Monica L. Bennett, Vibhas Deshpande, Ravi Teja Seethamraju, Emily Stern

**Affiliations:** 1 Department of Psychology, Northeastern University, Boston, Massachusetts, United States of America; 2 Functional Neuroimaging Laboratory, Departments of Psychiatry and Radiology, Brigham and Women's Hospital/Harvard Medical School, Boston, Massachusetts, United States of America; 3 Department of Psychology, University of Massachusetts, Amherst, Massachusetts, United States of America; 4 MR Research & Development, Siemens Healthcare, Boston, Massachusetts, United States of America; 5 MR Research & Development, Siemens Healthcare, Austin, Texas, United States of America; Massachusetts Institute of Technology, United States of America

## Abstract

It is well known that natural languages share certain aspects of their design. For example, across languages, syllables like *blif* are preferred to *lbif*. But whether language universals are myths or mentally active constraints—linguistic or otherwise—remains controversial. To address this question, we used fMRI to investigate brain response to four syllable types, arrayed on their linguistic well-formedness (e.g., *blif≻bnif≻bdif≻lbif*, where ≻ indicates preference). Results showed that syllable structure monotonically modulated hemodynamic response in Broca's area, and its pattern mirrored participants' behavioral preferences. In contrast, ill-formed syllables did not systematically tax sensorimotor regions—while such syllables engaged primary auditory cortex, they tended to deactivate (rather than engage) articulatory motor regions. The convergence between the cross-linguistic preferences and English participants' hemodynamic and behavioral responses is remarkable given that most of these syllables are unattested in their language. We conclude that human brains encode broad restrictions on syllable structure.

## Introduction

It is well known that natural languages share certain aspects of their design. For example, across languages, syllables like *blif* are preferred (e.g., overrepresented) relative to *lbif* [1]. While these typological facts are well established, their interpretation is controversial. One contentious issue concerns the status of language universals—whether they are myths [2], mere fossils of historical processes, or synchronic mental constraints that are active in the brains of all humans. To the extent such constraints are identified, a second question arises concerning their origins—whether they emanate from universal linguistic principles [3], or from nonlinguistic mental forces [4]. And indeed, language structure is not arbitrary. Rather, favored linguistic structures tend to minimize generic computational costs and optimize auditory perception and articulation [5]. While such accommodation of functional pressures could be the hallmark of an adaptive biological system for language, its presence obscures the origin of language universals.

Our experiment addresses this challenge using neuroimaging methods. We reason that if the underrepresentation of certain structures across languages only reflects sensory-motor pressures (e.g., *lbif* is harder to hear[6], [7] and articulate[8]), then the costs associated with its encoding should tax sensory and motor brain sites. An alternative explanation attributes linguistic preferences to the language faculty itself. At the center of the language system is the grammar—a set of violable algebraic constraints that express tacit linguistic preferences (e.g., “avoid structure *lbif*”) [3]. To the extent those grammatical constraints are universal, then the ban on *lbif* should be evident in all speakers, even if the relevant structures (*blif* and *lbif*) are both unattested in their language. Moreover, unlike the competing sensorimotor explanation, the grammatical account predicts that the ill-formed structure (e.g., *lbif*) should differentially engage traditional language areas in the brain compared to its better-formed counterpart (e.g., *bnif*). Our investigation tests these predictions.

### Sonority restrictions on syllable structure

To explain our experimental manipulation, we must first consider in greater detail the restrictions on syllable structure. Across languages, syllables like *blif* are preferred (e.g., more frequent) relative to syllables like *bnif*, which in turn, are preferred to *bdif*; least preferred on this scale are syllables like *lbif* [9]. Linguistic research attributes this hierarchy to universal grammatical restrictions on sonority—a scalar phonological property that correlates with the loudness of segments [10]. Least sonorous are stop consonants (e.g., *b, p*), followed by nasals (e.g., *n, m*), and finally the most sonorous consonants—liquids and glides (e.g., *l,r,y,w*). Accordingly, syllables such as *blif* exhibit a large rise in sonority, *bnif* exhibits a smaller rise, in *bdif*, there is a sonority plateau, whereas *lbif* falls in sonority. The universal syllables hierarchy (e.g., *blif≻bnif≻bdif≻lbif*, where ≻ indicates preference) could thus reflect a grammatical principle that favors syllables with large sonority clines—the larger the cline, the better-formed the onset.

In line with this possibility, linguistic evidence has shown that this hierarchy correlates with syllable frequency across languages[9] and similar preferences are also seen experimentally in the behavior of individual speakers: as sonority distance decreases, participants tend to misidentify the syllable (e.g., misidentify *lbif* as the disyllabic *lebif* [9], [11]–[14]. These misidentifications are documented irrespective of whether the syllables are present [15] or absent in participants' language[9], [11]–[14], and even when auditory pressures are minimized (e.g., by using printed materials[11], [12]). These results imply an abstract grammatical process that repairs ill-formed syllables as better formed ones (e.g., *lbif*→*lebif*)—the worse formed the syllable, the more likely its repair, hence its misidentification. Misidentification, in this view, is the signature of broad grammatical restrictions that are potentially universal.

The behavioral results, however, cannot fully rule out nonlinguistic explanations for the findings. One possibility is that the misidentification of syllables like *lbif* might be caused by an articulatory failure. Although participants do not overtly utter the target, they might nonetheless attempt to do so subvocally, and their (failed) attempts may result in misidentification. In fact, the observed behavioral difficulty associated with the syllable hierarchy might not even originate from any single functional constraint—linguistic or otherwise. In this view, no single network of the mind/brain is sensitive to the syllable hierarchy. Rather, the monotonic increase in the costs of processing ill-formed clusters results from multiple disparate origins (e.g., auditory, articulatory, and lexical) that merely converge to form a monotonic function. For example, the best-formed syllable *blif* might be strongly favored for its grammatical structure, *bnif* might be favored (less strongly) for its lexical familiarity (e.g., similarity to *snif*), whereas the worst-formed structure *lbif* might be disfavored for its articulatory demands. The monotonic effect observed in behavior is an artifact of this conjunction. To address this possibility, we turn to evidence from functional magnetic resonance imaging (fMRI).

### The present fMRI experiment

Our experiment presented English speakers with four types of spoken monosyllables, arrayed according to their sonority profile. The best-formed syllable with a large sonority rise (e.g., *blif*) is attested in English, but the other three types—small rises, plateaus and falls (e.g., *bnif, bdif, lbif*) are not allowed in this language. Participants were presented with these four types of syllables, mixed with their disyllabic counterparts (e.g., *belif, benif, bedif, lebif*) in a syllable-count task, while their brain response was imaged using a sparse sampling fMRI protocol (to enable the presentation of auditory stimuli in relative silence [16], [17]). In accord with past behavioral findings, we expect that, as sonority distance decreases, participants should selectively exhibit greater difficulty (i.e., more errors) in the identification of monosyllables, but not their disyllabic counterparts. Our primary interest concerns the brain signatures of this effect.

If the syllable hierarchy reflects an active mental constraint, then one should expect it to modulate the hemodynamic response of individual speakers. Accordingly, there should be brain loci whose activation varies monotonically along the syllable hierarchy. And if this hierarchy is shared across languages, this brain response should be found despite no experience with most syllable types, and it should be selectively related to the structure of the monosyllables (but not disyllables).

Having linked language universals to brain response, we can next probe for its source. Given the uncertain links between brain activity and function [18], [19], in general, and the multiplicity of functions associated with language areas, specifically [20]–[23], such inferences remain tentative, and they are further tempered by several methodological limitations of our study—issues we consider along with the discussion of our results. Such limitations notwithstanding, localization can nonetheless offer general clues for functional origins. If the effect of syllable structure is solely due to (nonlinguistic) auditory and articulatory demands[6]–[8], then it should be limited to primary auditory cortex and motor regions, including articulatory motor areas (the lip, tongue and larynx areas in primary motor cortex [24] and supplementary motor area).

Localization can further adjudicate between competing linguistic explanations for the results. The hypothesis of universal grammatical rules asserts that the brains of all speakers share a common set of algebraic linguistic principles that constrain the structure of any syllable—irrespective of whether it is present or absent in one's language [25], [26]. Our present experiment tests this hypothesis by gauging the response of English speakers to syllable types that do not occur in English. Generalizations to unattested syllables, however, do not necessarily demonstrate the representation grammatical rules. On an alternative account, the advantage of well-formed syllables (e.g., *blif*) reflects not their algebraic grammatical structure but rather their similarity to familiar words (e.g., to *black*) [27], [28]

The localization of the hemodynamic response may help distinguish between these possibilities. If the advantage of the well-formed syllables reflects their similarity to familiar words stored in the lexicon, then it is likely to engage regions associated with lexical processing (e.g., posterior regions of the superior temporal gyrus and the superior marginal gyrus [29], [30]. Conversely, if language universals originate from shared grammatical constraints, then the effect of syllable structure might extend to traditional language areas (Broca's and Wernicke's area). Such a finding would open the possibility that language universals are active mental constraints of linguistic origin.

## Behavioral Results

**Figure 1:**
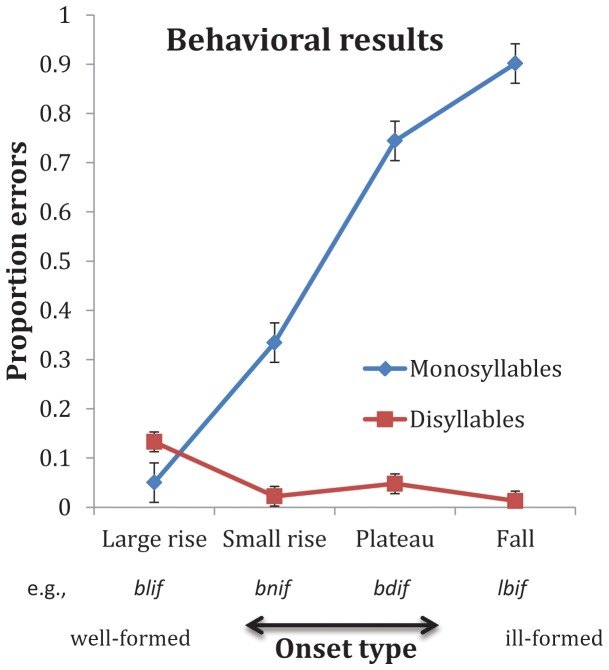
The effect of syllable hierarchy on behavior. As the stimulus became worse-formed on the syllable hierarchy, the proportion of errors increased selectively to monosyllables.

Figure 1 plots response accuracy as a function of syllable structure (In all figures, error bars are 95% confidence intervals constructed for the difference between the means). An inspection of the means suggests that monosyllables were harder to identify than disyllables, and identification accuracy varied monotonically with the structure of the syllable—as the syllable became worse formed, errors increased.

A 2 syllable (monosyllables vs. disyllables) x 4 type (e.g., *blif, bnif, bdif, lbif*) ANOVA on response accuracy (arcsine transformed), conducted using participants (F1) and items (F2) as random variables indeed yielded a reliable interaction (F1(3, 39) = 147.85, p<.0001; F2(3, 45) = 46.76, p<.0001).

A test of the simple main effect indicated that monosyllable type reliably modulated response accuracy (F1(3, 39) = 133.94, p<.0001; F2(3, 45) = 41.12, p<.0001). Planned comparisons further showed that monosyllables with large rises in sonority (e.g., *blif*) produced reliably more accurate responses relative to small rises (e.g., *bnif*, t1(39) = 8.68, p<.0001; t2(45) = 3.25, p<.003), which, in turn produced reliably more accurate responses compared to sonority plateaus (e.g., *bdif*, t1(39) = 7.14, p<.0001; t2(45) = 5.27, p<.0001); the contrast between sonority plateaus and falls (e.g., *lbif*) was marginally significant (t1(39) = 2.37, p<.03; t2(45) = 1.13, p<.28)

To demonstrate that the effect of syllable type is not due to artifact associated with binary data [31], we also submitted the results to a General Mixed Effects Model logistic regression model with syllable (monosyllables vs. disyllables) and type (e.g., *blif, bnif, bdif, lbif*) as fixed effects, and participants and items as random effects. The conclusions remained essentially unchanged. A comparison of monosyllables of adjacent sonority levels using forward difference coding showed that monosyllables with large rises in sonority (e.g., *blif*) produced reliably more accurate responses relative to small rises (e.g., *bnif*, β = 2.40, SE = 0.351, Z = 6.84, p<.0001), which, in turn produced reliably more accurate responses compared to sonority plateaus (e.g., *bdif*, β = 1.92, SE = 0.217, Z = 8.83, p<.0001). Finally, sonority plateaus produced significantly more accurate responses than falls (e.g., *lbif* β = 1.20, SE = 0.281, Z = 4.28, p<.0001).

These observations replicate past behavioral results [9], [11], [12], [32] showing that people are sensitive to the structure of syllables that they have never heard before. The subsequent fMRI analyses examine whether this pattern originates from a single source and investigate its origin.

## Imaging Results

Our analyses probed for the hypothesized 2 syllable (monosyllables vs. disyllables) x 4 type (large sonority rise, small rises, plateaus and falls e.g., *blif, bnif, bdif, lbif*), with a linear contrast of [−¾−¼ ¼ ¾]) interaction in the whole-brain voxel-wise ANCOVA conducted over the BOLD signal. We first tested the interaction in traditional language areas—Broca's (BA 44–45) and Wernicke's (BA 22) areas in the left hemisphere, along with their contralateral homologs. We next evaluated this interaction in three regions associated with speech processing, including primary auditory areas, motor areas and regions linked to lexical access (for definitions, see Method). The results are presented in Table 1.

**Table 1:** The effect of the syllable hierarchy on language areas (a), speech processing areas (sensorimotor and lexical, b) and other areas (c).

Contrast of Interest					2 Syllable x 4 Type Interaction					Monosyllables Linear Contrast					Disyllables Linear Contrast				
Brain Region					Cluster extent (mm^3^)	Peak z-score	Peak coordinate in MNI space (mm)			Cluster extent (mm^3^)	z-score at or near the corresponding peak coordinate	Corresponding coordinate in MNI space (mm)			Cluster extent (mm^3^)	z-score at or near the corresponding peak coordinate	Corresponding coordinate in MNI space (mm)		
							x	y	z			x	y	z			x	y	z
**a.**	***Language***	**L**	**45**	**P. Broca's**	**108**	**g00**	**−54**	**30**	**12**	**54**	2.55*	**−54**	**27**	**6**		**−** ***0.49***	**−** ***54***	***30***	***12***
		**L**	**45**	**A. Broca's**	**27**	**g00**	**−36**	**45**	**12**		**−** ***1.61***	**−** ***36***	***45***	***12***		***1.48***	**−** ***36***	***45***	***12***
		**R**	**45**	**P. Broca's**	**54**	**g00**	**54**	**30**	**9**	**1161**	2.84*	**51**	**30**	**18**		**−** ***0.93***	***54***	***30***	***9***
		**R**	**45**	**A. Broca's**	**27**	**g00**	**45**	**33**	**30**		**−** ***1.52***	***45***	***33***	***30***		***1.85***	***45***	***33***	***30***
**b.**	*Auditory*	L	41	TMA	27	3.31**	−36	−36	18	108	3.47**	−36	−33	15		−2.74*	−*39*	−*39*	*21*
		R	41	TMA		2.54*	*45*	−*27*	*18*		*2.15*	*45*	−*27*	*18*		−*2.25*	*45*	−*27*	*18*
	*Motor*	L	4	PC (Lip)		−3.02*	−*48*	−*12*	*39*		−2.43*	−*51*	−*12*	*39*		2.9*	−*48*	−*15*	*39*
		L	44	PC (Larynx)	81	−3.68**	−30	−3	27	54	−3.41**	−30	−6	27		3.07*	−*33*	*-3*	*24*
		R	3	PC (Larynx)	540	−3.91***	45	−15	39	27	−3.35**	39	−18	30		2.68*	*45*	−*9*	*39*
		L	8	SMA		2.75*	*0*	*21*	*63*		*1.96*	*0*	*21*	*63*		−*1.53*	*0*	*21*	*63*
	*Lexical*	L	42	SMG/pSTG		*2.22*	−*60*	−*24*	*18*		2.8*	−*60*	−*18*	*18*		−*0.39*	−*60*	−*18*	*18*
**c.**	*Other*	R		Cerebellum (Vermis 6)	135	3.81***	6	−57	−21		*0.92*	*6*	−*57*	−*21*		−3.07*	*6*	−*57*	−*21*
		R	27	Lingual	108	3.73***	15	−33	0		*0.14*	*15*	−*33*	*0*		−2.79*	*18*	−*33*	*0*
		L		Thalamus	108	−4.39***	−18	−9	3		−2.92*	−*18*	−*9*	*3*		2.59*	−*21*	−*9*	*3*
		L	7	S. Parietal	243	−4.12***	−24	−51	72		−2.9*	−*21*	−*54*	*75*	513	4.02***	−27	−48	72
		R	19	M.Occipital	486	−3.71***	39	−78	9		−2.62*	*39*	−*75*	*9*		*0.86*	*39*	−*78*	*9*

Note. For the hypothesized language regions (a), significance threshold is p<.05 (corrected); for the nonlinguistic speech regions (b), p<.001 (uncorrected); for all other regions (c), p<.0001 (uncorrected). Cluster extent for language areas is calculated at the .01 significance levels; all other clusters are calculated at the .001 level. All subthresholding trends are listed in italic font, for the purpose of showing the sign/direction of the activations. BA = Brodmann area; L = left hemisphere; R = Right Hemisphere; P = Posterior; A = Anterior, S = Superior; M = Middle; TMA  =  Transverse Temporal Gyrus; PC  =  Postcentral Gyrus; SMA  =  Supplementary Motor Area; pSTG  =  posterior Superior Temporal Gyrus; SMG  =  Supra Marginal Gyrus; *−p<.01; **−p<.001; ***−p<.0001.

### Language areas

**Figure 2:**
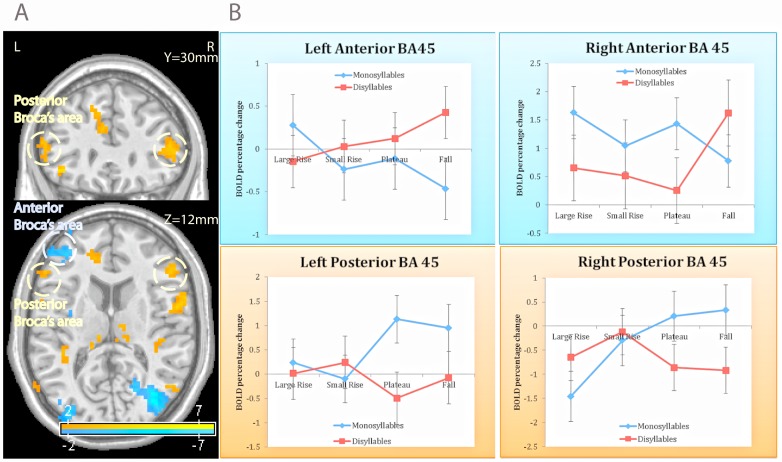
The effect of the syllable hierarchy on Broca's area. The syllable structure manipulation activated posterior Broca's area, but deactivated its anterior region (A). An inspection of the BOLD signal (B) showed that these changes were monotonically linked to the well-formedness of the monosyllables, but not their disyllabic counterparts. Responses to monosyllables are plotted in blue; disyllables are indicated in red.

The critical interaction was reliable in Broca's area (BA 45) bilaterally, but not in BA 44 or Wernicke's area. In each hemisphere, there were two peaks of activation—lateral posterior and an anterior (see Figure 2A and Table 1; to illustrate the spatial extent, in this and all other figures, clusters are shown at an initial voxel-wise p-value<.05). At the lateral posterior peak, grammatical ill-formedness triggered increase in BOLD signal (i.e., positive interaction term, significant bilaterally) whereas the anterior peak exhibited a decrease (negative interaction term, significant bilaterally).

These symmetric hemodynamic patterns could reflect two distinct consequences of grammatical well-formedness, whereby ill-formed syllables both incur a processing cost, and disengage the language system. If this interpretation is correct, then these effects should be (a) distinct for monosyllables and disyllables, and (b) monotonically related to syllable structure (e.g., *blif≻bnif≻bdif≻lbif*).

To evaluate these predictions, we plotted the changes in the BOLD signal observed at those sites relative to rest (we chose not to use disyllables as the baseline as their identification is demonstrably modulated by the sonority of their monosyllabic counterparts [9], [14], [32]). An inspection of these plots (see Figure 2B) suggests that the hemodynamic response was monotonically linked to the structure of the monosyllable.

Considering first the posterior sites, as the syllable became worse formed, activation selectively increased for monosyllabic stimuli, but not for their disyllabic counterparts, and these patterns emerged consistently across the two hemispheres. In addition, the worst formed monosyllables of falling sonority (e.g., *lbif*) elicited stronger activation than their (well-formed) disyllabic counterparts (e.g., *lebif*).

Tests of the simple main effect of syllable type in the ANCOVA confirmed that, at the posterior sites, syllable type reliably increased the activation for monosyllables at both the left and right hemisphere (initial p<.05, uncorrected), whereas for disyllables, this effect was negative and nonsignificant bilaterally (see Table 1).

The left anterior site yielded the mirror-image pattern. As syllable type became worse-formed, there was a monotonic decrease in activation for monosyllables, but not their disyllabic counterparts. Neither trend, however, reached significance in the simple main effect analyses of the left or right hemispheres (initial p<.05, uncorrected). The left anterior site also exhibited a decrease in activation for the worse-formed syllables of falling sonority relative to their disyllabic counterparts (Table 1).

Together, these results suggest that the ill-formedness of monosyllables results in two distinct hemodynamic responses in Broca's area: a posterior bilateral increase in activation, possibly due to the greater processing cost of ill-formed structures, and an anterior left-hemisphere deactivation, suggestive of disengagement.

### Sensorimotor/lexical areas

While syllable type modulates activation in Broca's area, it is conceivable that its effect might extend to other key regions mediating speech processing—auditory, articulatory and lexical. An inspection of the ANCOVA results indeed yielded significant type x syllable interaction in primary auditory area along with motor areas linked to the lip and larynx.

#### Primary auditory cortex

**Figure 3:**
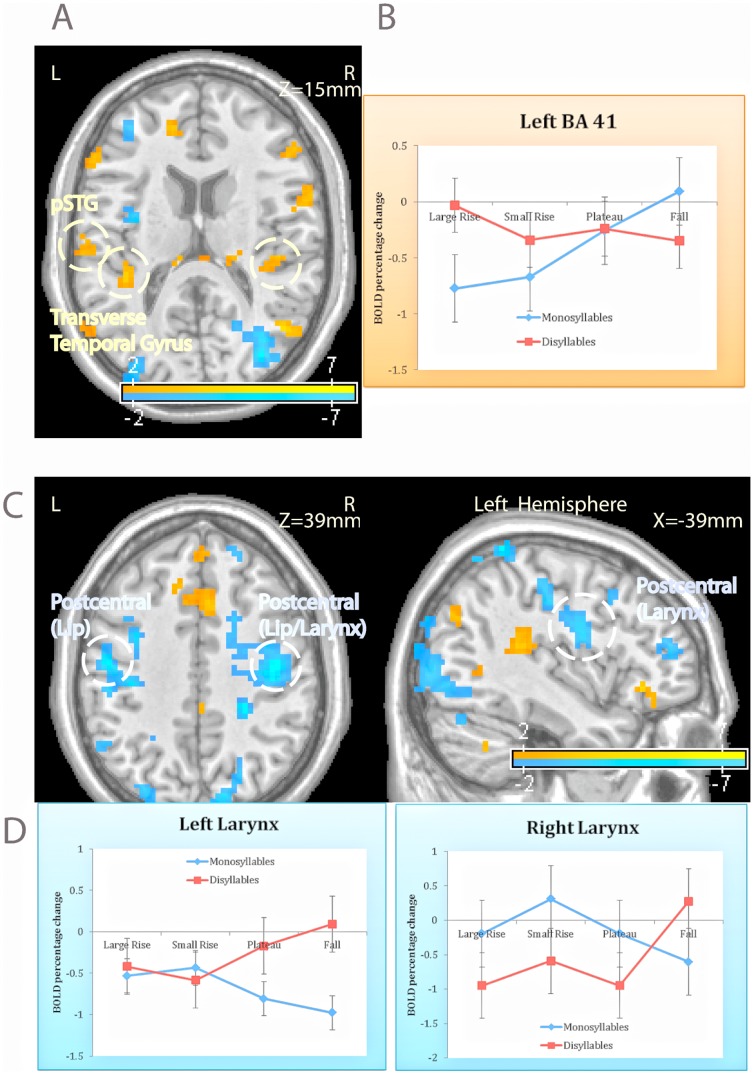
The effect of syllable hierarchy in sensorimotor speech areas. The syllable structure manipulation activated primary auditory cortex (A), and this effect was specifically due to the structure of monosyllables (B). Syllable structure also modulated hemodynamic response in motor areas (C), but these effects, significant at the larynx area, resulted in deactivation (D). Responses to monosyllables are plotted in blue; disyllables are indicated in red.

The ANCOVA yielded a reliable interaction at a site adjacent to left Heschl's gyrus (BA 41); a similar nonsignificant trend was also evident contralaterally. An inspection of the BOLD responses (relative to rest, see Figure 3A,B) suggested that ill-formed monosyllables significantly increased the hemodynamic responses (see Table 1b), whereas their disyllabic counterparts showed a nonsignificant deactivation.

#### Articulatory motor areas

Articulatory demands might present another explanation for the difficult encoding of ill-formed syllables. Although our task did not elicit overt articulation, the identification of spoken words could activate articulatory motor areas—both primary and supplementary motor areas. We thus asked whether ill-formed monosyllables tax those sites.

Considering first primary motor areas, the ANCOVA yielded a significant bilateral interaction at a site identified as a primary motor larynx site [24]. A similar, nonsignificant trend also emerged at the left lip area (an area that is lateral and superior to the larynx area, although the two areas are adjacent/overlapping [24]). In both cases, however, ill-formed monosyllables were associated with deactivation, rather than activation (see Figure 3C,D). Tests of the simple main effects of onset type (see Table 1b) suggested that ill-formed monosyllables reliably decreased activation in the larynx area (bilaterally), whereas their disyllabic counterparts resulted in a nonsignificant increase in activation.

The ANCOVA also yielded a marginally reliable onset x syllable interaction at a left supplementary motor site, but tests of the simple main effects (Table 1b) suggested that this interaction was solely due to the disyllables. Specifically, disyllabic counterparts of ill-formed onsets (e.g., *lebif*) tended to disengage this site compared to the counterparts of well-formed monosyllables (e.g., *benif*). Onset type, however, did not reliably modulate response to monosyllables.

#### Lexical interface

A third explanation for the misidentification of ill-formed syllables appeals to lexical analogy. In this view, well-formed syllables are better identified because they benefit from the activation of similar syllables, stored in the mental lexicon (e.g., *bnif* activates *sniff*). Our manipulation yielded some evidence of activation in the posterior part of the superior temporal gyrus and the superior marginal gyrus, but the relevant interaction did not reach significance in the ANCOVA. Moreover, the analyses of the simple main effects found no significant effect of syllable type for monosyllables (p>.001; see Table 1b).

### Other areas

Our manipulation of syllable type also yielded a reliable interaction in several other regions (see Table 1c). Most of those sites, however, showed a negative interaction term.

## Discussion

Our experiment was designed to investigate the encoding of language universals in the human brain. We first asked whether linguistic structures that are dispreferred across languages differentially engage the brain relative to preferred structures. A second goal was to shed light on the source of this effect—whether it solely stems from the auditory and articulatory demands associated with processing ill-formed variants and their similarity to familiar words, or whether it could also reflect their abstract grammatical structure.

Our results address both questions. Concerning the first, we found that the universal hierarchy of syllable structure (e.g., *blif≻bnif≻bdif≻lbif*) significantly modulated the hemodynamic response, and its shape closely mirrored the behavioral findings. Syllables that are dispreferred (e.g., underrepresented) across languages (e.g., *lbif*) were harder to identify—the worse formed the syllable, the more errors it produced, and such ill-formed monosyllables were also harder to identify than their disyllabic counterparts (e.g., *lebif*).

The hemodynamic response closely matched the effects seen in behavior. But unlike the behavioral findings, the hemodynamic pattern acquired two distinct manifestations. While some regions were activated by ill-formed syllables (i.e., an increase in hemodynamic response to ill-formed monosyllables relative to well-formed monosyllables and disyllables), other sites exhibited deactivation. These mirroring hemodynamic patterns could reflect two distinct consequences of the syllable structure. Ill-formed syllables (e.g., *lbif*) might impose greater processing demands (linguistic, or otherwise—an issue we discuss next), hence, become dispreferred. The patterns of activation and deactivation might reflect processing costs and preference, respectively. This last inference requires some caution, as our analysis does not effectively link the hemodynamic response to the cognitive representation of the stimulus at any particular trial (e.g., we do not know whether the brain response to *lbif* differs in a trial in which it is misidentified relative to one in which it is identified correctly). Nonetheless, across trials, the behavioral pattern was closely associated with two conflicting hemodynamic responses—activation and deactivation, respectively. In both cases, the effect of syllable structure was systematic, and it obtained relative to either well-formed monosyllables or well-formed disyllables. These results are remarkable because most of these syllables do not exist in English. These findings show for the first time that human brains are sensitive to putative universals concerning the sound structure of language (i.e., phonology).

Our findings also shed some light on the source of this sensitivity. Modern phonological theory has underscored the close links between linguistic phonological preferences and their sensorimotor processing demands[5]. Indeed, well-formed structures (e.g., consonant-vowel syllables, e.g., *ba*) tend to optimize sensorimotor transmission[33]. Nonetheless, the link between well-formedness and sensorimotor pressures is indirect, as the grammatical ban on ill-formed structures reflects not sensorimotor constraints (e.g., “reduce articulatory effort”), but (violable) rules (e.g., syllables must begin with a consonant) [3]. Thus, phonological rules are grounded in the sensorimotor system, but autonomous from it.

Several of our findings are consistent with this proposal. In accord with the grounding hypothesis, our results revealed that syllable structure modulated activation in several primary sensorimotor areas, including primary auditory cortex and primary motor cortex (bilaterally)—in an area that matches the larynx site [24]. The engagement of articulatory motor areas is noteworthy given that our task did not elicit overt articulatory response. The finding is consistent with a large literature demonstrating that perceptual cognitive tasks engage action networks [34], [35]. However, ill-formed structures did not invariably tax the hemodynamic response. While ill-formed syllables tended to activate primary auditory cortex, the opposite trend was evident in primary motor sites. Here, ill-formed syllables decreased activation, whereas their disyllabic counterparts exhibited an increase (probably because the disyllabic counterparts of sonority falls all begin with a sonorant consonant—a segment whose production engages the larynx, e.g., *lebif*). The deactivation of the larynx by monosyllables is inconsistent with the possibility that the misidentification of ill-formed syllables only reflects difficulties in their articulation.

Our results also yield no evidence that the difficulty in processing ill-formed syllables is due to their dissimilarity to familiar English words. While the locus of lexical phonological processing has been subject to debate[36], [37], parametric manipulations of factors related to lexical activation (e.g., word frequency, density, and familiarity[29], [30]) have implicated the left posterior superior temporal gyrus, left posterior temporal gyrus and the left suprmarginal gyrus in lexical processing, and related research [38] has demonstrated their engagement in the processing of stimuli that are similar to English syllables (e.g., *sli*). These areas, however, were not significantly engaged by our manipulation. It is possible that this null effect could stem from the choice of our experimental task and from power limitations of our statistical analyses, and as such interpretation requires caution. Nonetheless, this null effect is significant given the positive activation we had found in traditional language areas.

Specifically, our findings revealed systematic links of grammatical well-formedness to two sites at Broca's area (BA 45) and their right-hemisphere homologs. At the posterior site, ill-formed monosyllables increased the BOLD signal relative to either better-formed monosyllables or disyllables, whereas the anterior site yielded a deactivation pattern. Given the complex architecture of Broca's area [39] and the multiplicity of its presumed functions—both linguistic grammatical computations [20], [21], [40] and numerous nonlinguistic ones (e.g., mirroring action[22], cognitive control [23], and storage [41]), the precise functional explanation of this finding is not entirely clear.

One possibility is that the activation of Broca's area reflects domain-general demands associated with the controlled processing of these spoken stimuli. For example, the engagement of Broca's area might reflect difficulties in the controlled processing of ill-formed syllables. And indeed, ill-formed monosyllables are confusable with their disyllabic counterparts, and they engage primary auditory cortex to a greater extent than better-formed syllables. Given that BA 45 has been previously implicated in the deliberate processing of phonological information [42], the increase in activation might reflect the effects of acoustic costs on decision or the generation of response, rather than grammatical linguistic computations. While this explanation would seem to account for certain aspects of the results, the patterns of activation in posterior Broca's area and primary auditory cortex do not fully match. Compared to disyllables, ill-formed monosyllables increased activation in posterior Broca's area, but this effect was not seen at the primary auditory site.

Another nonlinguistic explanation attributes the involvement of Broca's area to motor processing, as the activation of the anterior Broca's site and its homolog closely matched the deactivation of primary motor sites, most notably, the left larynx. Similar deactivation was also evident in several components of the reading network (the superior parietal, middle occipital and BA 6, see Table 1c) [43], possibly because participants disambiguated the spoken inputs by generating their orthographic forms. But this account fails to explain why the deactivation of these sites (presumably, due to a decrease in processing cost) led to the increase in identification costs observed behaviorally.

On an alternative grammatical explanation, ill-formed syllables are dispreferred because they violate a set of grammatical constraints that are shared across languages, perhaps even universally. The violation of linguistic constraints would render these ill-formed syllables harder to encode by the language system, hence, dispreferred. The conflicting hemodynamic responses in Broca's area (activation vs. deactivation) could reflect the distinct consequences of grammatical ill-formedness. The view of phonological rules as grounded in the sensorimotor system further explains why ill-formed structures modulated auditory and motor sites, albeit in an indirect manner. Such modulation, in fact, could signal the role of these areas in grammatical phonological computations, not only in sensation and action. Our present results cannot settle the battle for Broca's area [20], and the results from English speakers may not apply universally. Nonetheless, findings that the syllable hierarchy systematically modulates brain activity, and applies to syllable types that participants have never heard before, suggest the existence of shared mental restrictions on syllable structure. These results open up the possibility that language universals are neither myths nor historical relics. Rather, they might reflect broad principles that are active in the brains of individual speakers and mirror their behavior.

## Methods

### Participants

Fourteen native English speakers took part in the experiment (10 females). They were all young adults (M = 22.57 years), right handed (as determined by the Edinburgh handedness inventory questionnaire), and they reported no hearing, neurological or psychiatric problems. Participants were paid $75 for their participation in the experiment. Informed written consent was obtained from all participants. This study was approved by the IRB at Brigham and Women's hospital and Northeastern University. Written informed consent was obtained from all participants.

### Materials

The experimental materials consisted of a set of 16 quartets of monosyllables (e.g., *blif, bnif, bdif, lbif*) along with their disyllabic counterparts (e.g., *belib, benif, bedif, lebif*), sampled from the materials used in previous research [9], [32]. Monosyllables were CCVC sequences (C = consonant, V = vowel) with a consonant cluster—either one with a large sonority rise, a small sonority rise, sonority plateau or sonority fall (e.g., *blif, bnif, bdif, lbif*). Except monosyllables with large rise, all monosyllables are unattested in English. Corresponding disyllables have the structure C∂CVC (e.g., *b∂lif, b*∂*nif, b*∂*dif, l*∂*bif*). The entire set of experimental materials is provided in Table S1.

The materials were recorded by a native Russian speaker (because these monosyllables are all possible in this language, they could be produced naturally by the speakers). These items were divided into four experimental runs (32 stimuli, balanced for the syllable x onset combinations), presented to each participant in four counterbalanced blocks with trial order randomized. Prior to the experimental session, participants were given practice consisting of 8 auditory words (with feedback).

### Procedure

The NNL fMRI Hardware System (NordicNeuroLab, Bergen, Norway) with E-Prime2.0 Professional software (Psychology Software Tools, Inc., Sharpsburg, PA, USA) were configured and programmed for sensory stimulus delivery and response recording that were synchronized with a Siemens MAGNETOM TIM Trio 3-Tesla MRI scanner (VB17A) (Siemens Medical Solutions, Erlangen, Germany), equipped with a standard 12-channel head coil. The fMRI experiment was conducted with a tailored scanning protocol with two anatomical image acquisitions, and a series of fMRI runs using a modified gradient echo EPI sequence that allows one to insert periods of “silent” time in the pulse sequence. The auditory stimuli were presented only during the predetermined “silent” gaps in the acquisition chain [17] and synchronized with the auditory stimulus presentation via E-Prime and NNL fMRI Hardware System.

Each fMRI experimental run started with a 25.9 second rest period (during which a fixation cross was presented), and was followed by 32 consecutive event-related trials, each of which lasted 13.2 seconds. Each experimental trial began with a visual cue, consisting of a sound icon, presented for 0.5 second. This cue was followed immediately by the presentation of the auditory stimulus within a silent scanning period of 1.2 second (corresponding to the length of the TR [repetition time; a single functional scan acquisition time]). This was synchronized with the silent steady state sampling scheme (described below). During the inter-stimulus interval from the end of the cue in trial *n* to the beginning of the cue in trial *n+1*, a fixation cross was displayed for 12.7 seconds—a period during which participants responded by pressing one of two buttons using their left hand (index finger  = 1 syllable; thumb  = 2 syllables). Each fMRI experimental run ended with a 22.8 second rest period.

### Image acquisition and analysis

#### MRI Image Acquisition

Images were acquired with a Siemens MAGNETOM TIM Trio 3-Tesla MRI scanner (VB17A) (Siemens Medical Solutions, Erlangen, Germany), equipped with a standard 12-channel head coil.

#### Structural imaging

Following a standard T1 weighted localizer scan, a high-resolution T1 weighted anatomical image was acquired using an MPRAGE acquisition sequence (TE/TR = 2.32/1900 ms, flip angle = 9°, 208 coronal slices with thickness  = 0.9 mm, field of view  = 240×187.2×240 mm^3^, voxel resolution  = 0.9375×0.9×0.9375 mm^3^). The T1 weighted MPRAGE image was then used to define the field of view and slice placement for functional imaging, via reformatting a set of 160 1 mm transverse slices parallel to the AC-PC line in the sagittal view and to set the transverse slice placement parallel to the line through the top of the left and right amygdalae in the coronal view. A reference T1 weighted anatomical image with the same axial slice placement and equivalent slice thickness as the functional imaging is then acquired (TE/TR = 12/600 ms, flip angle = 90°, 21 transverse slices with thickness = 3 mm and gap = 3 mm, field of view = 180×240 mm^2^, 384×512 matrix size, voxel resolution  = 0.46875×0.46875×6 mm^3^).

#### Functional imaging

Blood Oxygenation Level-Dependent (BOLD) contrast imaging was performed using a modified gradient echo EPI sequence that allows one to insert periods of “silent” time in the pulse sequence. When the “silent” mode is active, minimal residual background noise is achieved by eliminating the readout gradients and data acquisition triggers but keeping normal RF pulses and slice selective gradients to maintain the magnetization steady state (TE/TR = 30/1200 ms, flip angle = 70°, 21 5 mm transverse slices with 1 mm gap, field of view = 240×240 mm^2^, 64×64 matrix size, resulting resolution = 3.75×3.75×6 mm^3^). The auditory stimuli are presented only during the predetermined “silent” gaps in the acquisition chain [17] and synchronized with the auditory stimulus presentation via E-Prime and NNL fMRI Hardware System.

#### Functional Image Processing

The functional image processing pipeline consisted of the following steps using customized SPM software [44], [45] carried out on an UNIX server (Sun Microsystems, Oracle Corporation, Redwood Shores, CA): Manual AC-PC re-orientation of the two anatomical images with application of the transformation parameters of the reference T1 image to all the functional EPI-BOLD images; Realignment to correct for slight head movement between functional scans based on intracranial voxels; Co-registration of functional EPI-BOLD images to the corresponding high-resolution T1 MPRAGE anatomical image, based on the rigid body transformation parameters of the reference T1 image to the high-resolution T1 image for each individual subject; Stereotactic normalization to a standardized coordinate space (Montreal Neurologic Institute (MNI) version of Talairach space) based on the high-resolution T1 MPRAGE anatomical image to normalize for individual differences in brain morphology, and application of the normalization transformation to all functional EPI-BOLD images; Spatial smoothing of all the normalized functional EPI-BOLD images with an isotropic Gaussian kernel (full width at half maximum = 7.5 mm).

#### Functional Image analysis

A two-level whole-brain voxel-wise linear random-effects model was utilized to examine the effect sizes of the key Group/Condition contrasts in an ANCOVA setting. First, a voxel-wise multiple linear regression model was employed at the individual subject level. This was comprised of the regressors of interest, which consist of the stimulus onset times convolved with a prototypical hemodynamic response function, and the covariates of no interest, which consist of the temporal first-order derivative of the principal regressors (to compensate slight latency differences in individual hemodynamic response from the prototypical response function), global fluctuations, realignment parameters, and scanning run periods. Temporal filtering was performed to counter the effects of baseline shifts and higher frequency noise (than prototypical hemodynamic response), and an AR(1) model of the time course was used to accommodate temporal correlation in consecutive scans.

The effect at every brain voxel was estimated using the EM (expectation maximization) algorithm, and regionally specific effects were then compared using linear contrasts. That is, for each subject, the effect image for each condition was calculated, and was also combined in a series of linear contrasts to be entered into the second level group analysis to assess within-group effect sizes of the key hypotheses. Second, at the group level, a random-effects model was used (with the Subject factor as the random-effect), which accounts for inter-subject variability. The within-group effects of the predetermined hypothesis-driven contrasts were then estimated using an EM algorithm, with demographic variables (age, gender) incorporated as covariates of no interest. These group-level effect estimates generate statistical maps of the t-statistic, and the statistical significance of the t-maps were thresholded at an initial voxel-wise p-value <0.01.

The fMRI imaging data processing procedures was performed using laboratory optimized Statistical Parametric Mapping (SPM) software [44], [45], and a whole-brain voxel-wise multi-level random-effects model in an ANCOVA setting was estimated to detect activation and deactivation patterns in BOLD signal with particular focus on pre-determined contrasts examining the effects of syllable x type interaction. Based on random field theory as implemented in SPM, the p-values at the peak voxels within the language areas of interest (Broca's (BA 45) and Wernicke's (BA 22) areas in the left hemisphere, along with their contralateral homologs) were corrected based on family-wise error rate over a sphere with a radius  = 3 mm which results in a search volume of 113 mm^3^  = 0.1 resel, and the t-stat at a peak voxel within an ROI was considered statistically significant if the corrected p-value <0.05. For additional key sensorimotor/lexical regions, voxel-wise p<0.001 (uncorrected), spatial extent >108 mm^3^. For all other areas, we adopt a voxel-wise threshold of p<0.0001 (uncorrected).

#### The definition of regions of interest

Broca's area (BA 44–45) and Wernicke's area were identified according to the standardized, anatomically-parcellated brain atlas developed by Tzourio-Mazoyer et al., (2002) [46]. For nonlinguistic regions, we used the coordinates from previous published research to guide our probing of the areas of interest. Specifically, primary auditory area was defined by the coordinates provided in Engelien et al. (2006) [47] (Table 1), whereas Motor areas (the lip, larynx and tongue) were defined according to Brown et al, (2008) [24] Table 2. For the lexical interface, we probed our data against the coordinates provided by multiple sources, including Graves et al. (2008)[48] (Table 1); Gow et al. (2008) [49] Table 1; and Prabhakaran et al., (2006) [29] Table 2a. None of these lexical sites were significant in our results.

## Supporting Information

Table S1 The experimental materials.
(DOCX)
