# Supplementary material for: Language Universals Engage Broca's Area
Source: PLoS One. 2014 Apr 17;9(4):e95155. doi: 10.1371/journal.pone.0095155 (PMC3990587; doi:10.1371/journal.pone.0095155)
Supplement: Table S1 — The experimental materials. (DOCX) [file pone.0095155.s001.docx]

**Table S1**. The experimental materials

| **Large rise** | **Small rise** | **Plateau** | **Fall** |
| --- | --- | --- | --- |
| Blif | bwif | bdif | lbif |
| drif | dlif | dbif | rdif |
| drɑf | dlɑf | dgɑf | rdɑf |
| dwʊp | dmʊp | dgʊp | mdʊp |
| drʊp | dnʊp | dbʊp | rdʊp |
| grɛf | gmɛf | gbɛf | rgɛf |
| gwit | gmit | gbit | mgit |
| kræf | kmæf | kpæf | rgæf |
| klʊp | kmʊp | ktɑp | ltɑp |
| trʊf | tlʊf | tkʊf | rtʊf |
| twɛp | tlɛp | tkɛp | mtɛp |
| trɑk | tnɑk | tkɑk | rtɑk |
| twæf | tmæf | tpæf | mtæf |
| trɛf | tnɛf | tpif | rtɛf |
| twʊk | tnʊk | tgʊk | mgʊk |
| træp | tmæp | tpæp | rpæp |
